# Is cesarean scar defect becoming history? The effect of uterotomy closure

**DOI:** 10.1590/1806-9282.20241507

**Published:** 2025-05-02

**Authors:** Neset Gumusburun, Ulya Uskent

**Affiliations:** 1 Tokat Gaziosmanpasa University Hospital, Department of Obstetrics and Gynecology – Tokat, Türkiye.; 2 Gayrettepe Florence Nightingale Hospital, Department of Obstetrics and Gynecology – İstanbul, Türkiye.

**Keywords:** Niche, Caesarean section, Scar, Suture technics

## Abstract

**OBJECTIVE:**

Isthmocele or cesarean scar *defect is* a pouch-like defect in the myometrium at the isthmic level that *is thought that it might occur* as a result of the *insufficient* healing process of the uterine incision after cesarean section. It is important not to underestimate isthmocele and its preventive measures since it might cause serious gynecologic and obstetric complications. However, the best suturing technique suitable for the prevention of isthmocele formation is yet to be identified. The aim of this study was to compare the effects of three different uterine closure techniques applied during cesarean section on isthmocele formation.

**METHODS:**

In this prospective study, a total of 120 term (>37 weeks) pregnant women with no history of cesarean section and scheduled for primary cesarean section were *randomized* preoperatively *to three different* uterotomy closure *techniques (baseball,* single-*locked, and single-unlocked groups).*

**RESULTS:**

In a total of 43 patients, postoperative third-month sonography revealed isthmocele as an *anechoic triangular area with* ≥*1 mm* depth at the scar site. Compared with the single-locked and single-unlocked groups, isthmocele development was significantly lower in the baseball-type closure group (47.5% in the single-locked, 46.2% in the single-unlocked, and 15.4% in the baseball-type closure group). The group with the highest residual myometrial thickness, that is, 5.7 mm, was again the patients who underwent baseball sutures.

**CONCLUSION:**

Uterotomy closure with baseball-type suturing seems to be an advantageous method as compared to the traditional techniques in terms of preserving the residual myometrial thickness and preventing isthmocele formation.

## INTRODUCTION

Currently, the most common surgical procedure performed by obstetricians in women of reproductive age is the cesarean section (C/S)^1^. The increase in C/S numbers worldwide has become a global concern. Elective C/S has no proven feto–maternal benefits; on the contrary, it might result in adverse outcomes, such as cesarean scar defects (CSDs), bladder–bowel injuries, and intra-abdominal adhesions. CSD or isthmocele, also known as a niche, might occur as a result of the insufficient healing process of the uterine incision after C/S^2^. It is generally about a 1–2 mm pouch-like defect, leading to thinning of the anterior myometrium and may also lead to abnormal uterine bleeding, chronic pelvic pain, scar ectopic pregnancy, uterine rupture, placenta accreta spectrum disorders, and infertility^3^. The prevalence of isthmocele, which ranges between 19 and 84%, continues to increase worldwide with a decline in the vaginal birth rate^4,5^.

Being an easily accessible, simple, non-invasive, and inexpensive method, transvaginal ultrasonography (TVUSG) typically detects isthmocele as an anechoic triangular defect at the isthmic level. The diagnosis can also be made with other modalities, such as magnetic resonance imaging (MRI), hysteroscopy (H/S), hysterography (HSG), and sonohysterography (SIS).

Isthmocele can be managed medically (e.g., levonorgestrel-releasing intrauterine system and oral contraceptives) or surgically (e.g., laparotomy, laparoscopy, H/S, and robotic or vaginal surgery). However, there is no consensus on which patients should be treated and how. While it is generally preferred by experts to treat only symptomatic patients, there is insufficient information about the success of the surgical management of isthmocele and its complications and recurrence after the surgery in the long term. Therefore, it would be reasonable to focus on taking preventive measures against isthmocele formation.

A retroverted uterus can cause more tension and thus might negatively impact the healing process. The uterine position and individual/genetic predisposition to insufficient wound healing can be considered patient-specific risk factors for isthmocele formation.

The best suturing technique suitable for the prevention of isthmocele formation is yet to be identified. The aim of this study was to compare the effects of three different uterine closure techniques applied during C/S on isthmocele formation.

## METHODS

In this prospective study, a total of 149 pregnant women scheduled for primary C/S in the Gynecology and Obstetrics Department between March and September 2022 were evaluated. Informed consent was obtained from all participants. Only term (>37 weeks) pregnant women with no history of C/S who were planned to undergo a primary C/S were included in the study. The study exclusion criteria were as follows: presence of regular contractions and/or a cervical dilatation of more than 4 cm indicating the beginning of the active stage of labor, placental abnormalities, previous uterine surgery, multiple pregnancies, premature rupture of membranes, chorioamnionitis, preoperative hemoglobin level below 10 g/dL, body mass index (BMI) above 35 kg/m^2^, any comorbidities (e.g., diabetes, hypertension, preeclampsia, and eclampsia), smoking and/or alcohol use, and the need for blood transfusion.

A total of 29 patients were excluded from the study because 12 patients did not fulfill the criteria and 17 refused to participate in the study. Finally, 120 women were eligible for the study and randomized preoperatively to three different uterotomy closure techniques (baseball, single-locked, and single-unlocked groups). In all three groups, No. 1 absorbable multifilament polyglactin 910 (Vicryl, Ethicon Inc., Somerville, NJ, USA) suture thread was used to close the uterine incision. When necessary, hemostatic additional sutures were applied using the same material. The three different suture techniques applied during uterotomy closure are as follows:

***Group 1 (Baseball Suturing Technique)*:** A corner suture was placed at the right corner of the incision. Next, the second stitch was placed at the apex of the left corner and tied with a knot. Then, the free end of the suture was cut and running baseball stitch pattern was started. The suturing pattern was performed by taking bites from the inside out through the upper and lower lips of the wound at approximately 1 cm intervals with a 1 cm margin from the wound edges.

***Group 2 (Single-Layer Locked Continuous Suturing Technique)*:** A corner suture was placed at the right corner of the incision. Next, the second stitch was placed at the apex of the left corner and tied with a knot. Then, the free end of the suture was cut and single-layer-locked continuous suturing was started. The suturing pattern was performed by taking bites from outside to inside through the lower lip and inside to outside through the upper lip of the wound. Each time, a lock was formed by passing through the loop formed by the previous suture. The suturing was performed at approximately 1 cm intervals with a 1 cm margin from the wound edges.

***Group 3 (Single-Layer Unlocked Continuous Suturing Technique)*:** The uterotomy line was closed in a single-layer continuous suturing pattern that is explained above as group 2 but without passing the needle through the loop formed by the previous sutures.

Three months after the operation, any presence of isthmocele and its anatomical location were evaluated by ultrasonography. Any presence of isthmocele would reveal itself by an anechoic triangular area with ≥1 mm depth at the scar site. Postpartum complaints and other data regarding maternal age, gestational week, gravida, parity, number of abortions, BMI, C/S indications, type of anesthesia, birth weight, operation time, preoperative–postoperative hemoglobin values, and whether or not additional sutures required during uterotomy closure were also recorded.

### Statistical analysis

Based on power analysis, 37 patients in each group were required to assess statistical significance (power of 0.80 and α=0.05). The power calculation was based on residual myometrial thickness (RMT). The statistical power analysis program G Power software version 3.1.9.7 was used. Mean and standard deviations (SDs) were reported for the normally distributed continuous variables. Median and interquartile range (IQR/Q1–Q3) were obtained when the SD was greater than the mean. The Shapiro-Wilk test was used to evaluate whether the data were normally distributed. Frequencies and percentages were calculated for categorical variables and presented as n (%). Since the outcome was categorical/binary, comparisons between the three groups were made by the chi-square test. A "p-value" less than 0.05 was considered statistically significant. The analysis of all variables was done with the statistical software program R Studio 2022.07.2 Build 576.

### Ethical aspects of the research

Ethics committee approval was obtained from Tokat Gaziosmanpaşa University Hospital before the study (date: March 17, 2022/project no. 22-KAEK-057). The study was conducted in accordance with the Declaration of Helsinki and the ethical standards of our country.

## RESULTS

The demographic characteristics of the patients in the study are shown in Table 1.

**Table 1:** Demographic characteristics of the patients.

Variables		Baseball (n=39)	Locked (n=40)	Unlocked (n=39)
Age (years)		25.5±5	27.3±5.2	27.4±5.3
BMI (kg/m^2^)		28.5±4.9	29.3±4.5	30.4±5.8
Gravida		39 (100)	40 (100)	39 (100)
	1	23 (59)	24 (60)	24 (61.5)
	2	7 (17.9)	6 (15)	9 (23.1)
	3	5 (12.8)	4 (10)	3 (7.7)
	4	3 (7.7)	3 (7.5)	1 (2.6)
	5	0 (0)	1 (2.5)	1 (2.6)
	6	0 (0)	1 (2.5)	1 (2.6)
	7	0 (0)	0 (0)	0 (0)
	8	1 (2.6)	0 (0)	0 (0)
	9	0 (0)	0 (0)	0 (0)
	10	0 (0)	1 (2.5)	0 (0)
Parity		39 (100)	40 (100)	39 (100)
	Nulliparity	26 (66.7)	26 (65)	28 (71.8)
	Multiparity	13 (33.3)	14 (35)	11 (28.2)
	Grand multiparity (≥5)	0 (0)	0 (0)	0 (0)
Abortion		39 (100)	40 (100)	39 (100)
	Present	7 (17.9)	5 (12.5)	8 (20.5)
	Absent	32 (82.1)	35 (87.5)	31 (79.5)

Categorical data are presented as n (%) and numerical data are presented as mean±standard deviation. BMI: body mass index.

Obstetric data such as gestational week, indications for CS, and number of additional sutures used in the operation are shown in Table 2.

**Table 2:** ’Obstetric characteristics of the patients.

Variables		Baseball (n=39)	Locked (n=40)	Unlocked (n=39)
Gestational age (weeks)		37.9±5	38.4±1	38.9±1
Duration of operation (min)		25.9±5.5	24.4±5.8	24.7±5
Birth weight (kg)		39 (100)	40 (100)	39 (100)
	2,000–2,500	7 (17.9)	1 (2.5)	2 (5.1)
	2,500–3,000	8 (20.5)	17 (42.5)	10 (25.6)
	3,000–3,500	18 (46.2)	11 (27.5)	20 (51.3)
	3,500–4,000	5 (12.8)	9 (22.5)	7 (17.9)
	4,000–4,500	1 (2.6)	2 (5)	0 (0)
Indications for C/S		39 (100)	40 (100)	39 (100)
	Non-reactive NST	18 (46.2)	11 (27.5)	12 (30.8)
	Cephalopelvic disproportion	7 (17.9)	10 (25)	15 (38.5)
	Fetal distress	7 (17.9)	8 (20)	4 (10.3)
	Abnormal lie or presentation	7 (17.9)	11 (27.5)	8 (20.5)
Additional suture used		39 (100)	40 (100)	39 (100)
	Present	4 (10.3)	12 (30)	10 (25.6)
	Absent	35 (89.7)	28 (70)	29 (74.4)
Type of anesthesia		39 (100)	40 (100)	39 (100)
	Spinal	36 (92.3)	36 (90)	36 (92.3)
	General	3 (7.7)	4 (10)	3 (7.7)

Categorical data are presented as n (%) and numerical data are presented as mean±standard deviation. C/S: cesarean section; Non-reactive NST: non-reactive nonstress test

A total of 43 (36.4%) patients were found to have isthmocele on TVUSG performed at postpartum week 12. Compared with the single-locked and single-unlocked groups, isthmocele development was statistically significantly lower in the baseball-type closure group (p-value=0.004) (Table 3).

**Table 3:** Comparison of the three different suture techniques on isthmocele development.

Variables		Baseball (n=39)	Locked (n=40)	Unlocked (n=39)	p-value
Isthmocele					
	Present	6 (15.4)	19 (47.5)	18 (46.2)	0.004*
	Absent	33 (84.6)	21 (52.5)	21 (53.8)	

* p-value <0.05 indicates statistically significant difference, on comparison of all groups.

## DISCUSSION

Since the number of C/S is increasing over time, the rate of isthmocele and its complications are also increasing. This helps obtain wider data on this pathology. On the other hand, there is still no consensus on its definition since different sources provide different myometrial indentation values to meet the diagnosis^6^. Osser et al. emphasized that as the number of C/S increases, wound healing will be adversely affected due to the old scar tissue and thus the rate of isthmocele and its complications may also increase^7^.

When randomly selected patients with a history of C/S were evaluated by TVUSG, the prevalence of isthmocele was found to be between 24 and 70%^5^. A previous cohort study had indicated isthmocele prevalence to be 43.4%^8^. In our study, the isthmocele incidence was found to be compatible with the literature. Osser et al. reported that the incidence of isthmocele after the first C/S was 61%, after the second C/S 81%, and after the third C/S 100%^4^.

Bennich et al. proposed maternal age, uterine position, and induction of labor as risk factors for the formation of isthmocele. Studies have suggested four hypotheses regarding the etiology of isthmocele, mostly based on patient-specific and surgically induced factors. The first hypothesis is that cervical glandular structures impair healing when the uterine incision opening is too low. Two different studies confirmed this hypothesis by showing that the defect was located below the intact scar. The second hypothesis is related to impaired healing due to early adhesion development between the uterotomy scar and the anterior abdominal wall. A thinner inferior myometrial segment occurs after cervical effacement in advanced labor. This may result in less vascularization leading to isthmocele formation^5^. Ballopra et al. suggested that intraoperative cervical dilatation performed by obstetricians to reduce the tension around the cesarean incision by draining excess blood from the uterus was associated with a lower risk of isthmocele formation. An opposing view was that this might disseminate any existing vaginal infection to the surgical site. Any increase in the risk of endometritis and sepsis was not found in the mentioned study, nonetheless. The third hypothesis is related to the surgical technique that results in the uterine wall not closing completely. Therefore, hysterotomy closure technique is considered an important factor in scar healing. The differences in scar morphology have been described between single-layer and double-layer, locked and unlocked techniques. On the other hand, in a meta-analysis of nine studies, Di Spiezio et al. found no difference between single- and double-layer techniques in isthmocele incidence^9^. In a meta-analysis by Roberge et al. involving 5,810 women, similar results were reported between the groups in terms of isthmocele incidence; nonetheless, the advantages of the double-layer closure technique were emphasized^10^. Marchand et al. showed that double-layer sutures resulted in higher RMT without a lower isthmocele rate^11^. A meta-analysis of 20 studies by Stegwee et al. found that the single-layer-locked suturing technique was associated with lower RMT and an increase in isthmocele incidence^12^. In another study, Ceci et al. compared the single-layer-locked continuous technique with the single-layer-interrupted technique and reported that the locked technique was associated with a larger defect^13^. Bamberg et al. compared three different uterotomy closure techniques: continuous single-layer unlocked, continuous single-layer locked, and double-layer sutures. They observed that the isthmocele incidence and its depth were independent of these three techniques^14^. The uterine suturing technique seems to be an important factor during C/S, but it remains unclear which technique is best to reduce isthmocele formation. Our findings suggest that women with baseball-type uterine sutures are less likely to develop isthmocele than women with single-locked and unlocked sutures. According to our findings, it seems that surgeons can prefer baseball-type suturing during C/S to preserve the RMT and reduce isthmocele formation. The fourth hypothesis regarding isthmocele formation includes patient-specific factors, such as poor hemostasis, tissue ischemia, individual/genetic predisposition contributing to inflammation or adhesion formation, and impaired wound healing, which may influence the development of isthmocele.

### Limitations and strengths of the study

There are studies in the literature investigating the effect of surgical closure technique on isthmocele formation. Nevertheless, the effect of baseball suturing on isthmocele formation has never been studied in the literature so far. This feature might be evaluated as the strong side of our study. Our findings suggest that uterotomy closure with baseball-type suturing seems to be an advantageous method as compared to the traditional techniques in terms of preserving the RMT and preventing isthmocele formation. On the flip side, as limitations of our study, the power was 80% and the comparisons were made at postpartum third month. Therefore, further studies with 90% power investigating the long-term complications regarding isthmocele are still needed.

## CONCLUSION

It is important not to underestimate isthmocele and its preventive measures since it might cause serious short- and long-term complications. Our findings suggest that women with baseball-type uterine sutures are less likely to develop isthmocele than women with single-locked and unlocked sutures. According to our findings, it seems that surgeons can prefer baseball-type suturing during C/S to preserve the RMT and reduce isthmocele formation. Our results need to be supported by large-scale studies including long-term complications.

## Data Availability

The data that support the findings of this study are available from the corresponding author upon reasonable request.
